# A Proposed Scan Strategy Used on SLM Inner Structure Part

**DOI:** 10.3390/ma13040937

**Published:** 2020-02-20

**Authors:** Jitai Han, Meiping Wu, Weipeng Duan

**Affiliations:** 1 School of Mechanical Engineering, Jiangnan University, Wuxi 214122, China; hanjitai@hotmail.com (J.H.); weipengduan@126.com (W.D.); 2 Additive Manufacturing Products Supervision and Inspection Center of Jiangsu Province, Wuxi Institution of Supervision & Testing on Product Quality, Wuxi 214073, China; 3 Department of Mechanical, Aerospace and Biomedical Engineering, University of Tennessee, Knoxville, TN 37996, USA

**Keywords:** selective laser melting, Ti6Al4V powder, inner structure, surface quality, scan strategy

## Abstract

A model with an inner structure was designed to study the relationship between the surface quality of the inner structure and the scan strategy in this study. The test results showed that the precision of the inner structure was highly affected by the scan strategy, and the specimens printed using different strategies showed different performances on the surface quality of the inner structure. The specimen printed using the square-framed scan strategy had a lower flatness value on the positive face of the inner structure compared to that of the other two specimens printed using Z-shape scan strategies, while the specimen printed using the Z-shape scan strategy (along the inner structure) had a relative optimal surface roughness on the side surface of the inner structure in all three specimens. The bending deformation caused by the scan strategies was considered to be the main factor affecting the flatness on the positive surface, while laser energy fluctuation showed a significant impact on side surface roughness. Combined with the experimental data, a new scan strategy was proposed; we found that the specimen printed using this new strategy improved positive surface flatness and side surface roughness.

## 1. Introduction

Three-dimensional printing (3DP) has attracted increasing attention all over the world due to its near net-shape formability, which is both time- and cost-saving [1]. The selective laser melting (SLM) machine can print a metal solid part by melting metal powder using laser energy, and it has been widely used in space, aviation, automotive, and other industries [2]. According to previous studies, there are about 100 factors affecting the properties of the printed parts, but only about 20 of them are considered to be controllable, including layer thickness, hatch spacing, scan speed, scan strategy, laser power, and built orientation [3]. In this research, scan strategy was the only variable parameter due to its commonality and high impact on the properties of printed parts.

Scan strategy has been frequently studied in recent years. Cheng et al. [4] found that different scan patterns had a great impact on residual stress, and a 45°-line scan case led to less deformation in all their results. Lu et al. [5] studied different island scan strategies. They found that the sample printed using a 2 mm × 2 mm island scan strategy had the lowest residual stress, while the samples printed using 5 mm × 5 mm and 7 mm × 7 mm island scan strategies had higher relative densities. Mohanty et al. [6] proved that a cellular scan strategy was a reliable simulation method to generate an optimized scan strategy. Wan et al. [7] investigated the effect of scan strategy on grain structure and crystallographic texture. They found that, for the specimen printed using an XY-direction scan strategy, the competitive grain growth mechanism became more evident and a strong cube texture was developed. Ahrari et al. [8] developed a method for multiobjective optimization of the scan path in selective laser melting. They tried to optimize the scan strategy with a nondominating sorting genetic algorithm. The multiobjective evolutionary method was used to find a set of trade-off solutions for the defined conflicting objectives on a process simulation domain consisting of 32 cells, which could not be obtained by performing merely a local search. Wild et al. [9] reported that the scan strategy and laser parameters should be modified according to the part’s geometry. Open-porous NiTi scaffolds with porosity up to 88% and very dense lattice material (density >99.8%) were printed. Xie et al. [10] used a knit scan strategy and found that the porosity of the printed part was significantly decreased compared to the specimen printed using the single-direction scan strategy. Jhabvala et al. [11] showed that the parallel scan strategy had some drawbacks. It was better to use a multidirectional scan strategy to fill this gap. Parry et al. [12] tried to explain the effect of scan strategy using thermomechanical simulation. They found that the design of laser scan strategies should avoid a long scan vector length and the direction of scan vectors should be orientated uniformly to produce an isotropic stress field in the component. Rashid et al. [13] studied the effect of scan strategy on the density of 17-4PH part. They found that the density of the printed parts increased after printing twice on each layer. Ghouse et al. [14] found that scan strategy had a great impact on the porosity of the printed parts. To print a part with high density, they optimized their scan strategy and changed the process parameters accordingly.

The surface quality of the printed parts has also been studied in the last few years. Calignano et al. [15] investigated accuracy and dimension limitation in the printing process. They said that the printed part with a base size below 0.8 mm and a geometry with sharp edges could not be reached using aluminum alloy. Mumtaz et al. [16] studied the effect of laser parameters on surface roughness. They found that process parameters had a significant impact on top and side surface roughness. Tian et al. [17] mainly investigated the influence of process parameters (laser power, scan speed, layer thickness, and sloping angle of the surface) on surface roughness printed using the Hastelloy X alloy. They confirmed that contour and skywriting scan strategies can be helpful in reducing surface roughness. Bagheri et al. [18] found that some geometries and mechanical mismatches can be reduced using a compensation strategy in the process. For strut thickness, the results showed that the largest mismatch (60% away from the design) occurred for horizontal members, which reduced to 3.1% upon application of the compensation. In this work they also studied the surface quality of general objects. They captured the dependence of strut thickness on the building angle in a spider web, and this scheme has been successfully applied to Ti6Al4V three-dimensional lattices, with a cell topology suitable for load-bearing applications.

The main focus of the current study was the precision of the inner structure, which includes the surface quality of the side and positive surfaces of the inner structure part. This field has been studied much less than the mechanical properties of the printed parts. This is due to the fact that some postprocessing treatments can be employed after printing the general parts; however, as it is hard to postprocess the inner structure surface, especially some microinner structures, it is meaningful to optimize the precision of the inner structure in the printing process.

In this study, the relationship between the surface quality of the inner structure and the scan strategy was studied. The inner structure part had both an inner structure layer and a noninner structure layer, which was different from the studies listed before. According to the special structure characteristic of the inner structure part, a new scan strategy was proposed to improve the surface quality of the inner structure.

## 2. Material, Methods, and Procedure

**Figure 1:**
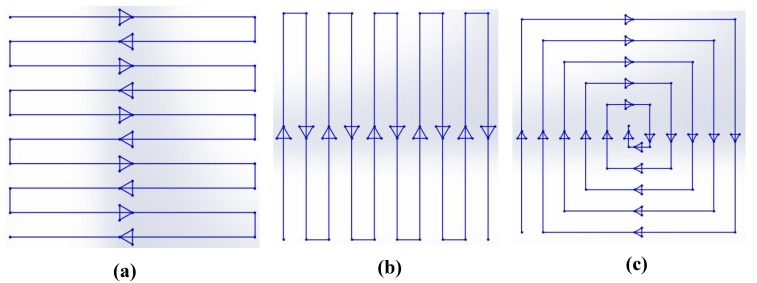
Three scan strategies used in this study. (**a**) Z-shape scan strategy vertical to the inner structure; (**b**) Z-shape scan strategy along the inner structure; (**c**) square-framed scan strategy.

**Figure 2:**
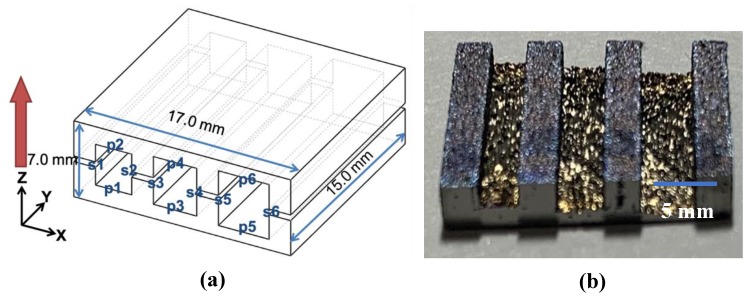
Specimen model with measuring information(**a**) and cutting surface(**b**) in this study.

The Ti6Al4V metal powder used in this research was supplied by Shenzhen Minatech Co., Ltd. (Shenzhen, China), and it was printed with dimensions of 17.0 mm × 15.0 mm × 7.0 mm (L × W × H) using FS271M (Farsoon, Changsha, China). Three different scan strategies were used, as shown in Figure 1. These were a Z-shape scan strategy vertical to the inner structure (in X direction (Figure 2a)) shown in Figure 1a; a Z-shape scan strategy along the inner structure (in Y direction (Figure 2a)) shown in Figure 1b; and a square-framed scan strategy (Figure 1c). The printed specimen in this study is shown in Figure 2. The positive and side surfaces of the inner structure performed differently, so they were studied separately. Therefore, P1–P6 were defined as the positive face, while S1–S6 were defined as the side face. The building orientation is also shown with the red arrow in Figure 2a.

The Trilinear Coordinates Measuring Instrument (Hexagon Metrology, Stockholm, Sweden) was used to test the surface flatness of the inner structure. Specimens were processed using a slow wire cutting machine, and the Surface Roughometer (Mitutoyo Kanagawa, Japan) was used to measure the roughness of the inner structure. A 3D surface profiler (RTEC, MFD-F profilometer 2207, San Jose, CA, USA) and a scanning electron microscope (SEM) (Carl Zeiss, Sigma300, Oberkochen, German) were also used to test the properties of the inner structure to give an explanation of the phenomenon. The detailed steps for the entire study are given below.

The first step of this experiment was to print the specimens using the three different scan strategies shown in Figure 1. Some other process parameters, such as laser power and layer thickness, were all set to an optimized condition according to our previous experiment, shown in Table 1. The diameter and chemical composition of the Ti6Al4V powder used in this study are listed in Table 2. The specimens were printed under the same conditions with the same Ti6Al4V powder, and all were printed in one base plate. After the printing process, the specimens were cut from the base plate using a slow wire cutting machine, provided by Wuxi Institute of Technology, to ensure precision.

The second step was to cut the specimen into two parts to measure the surface quality of the inner structure. The cutting machine and mode were similar to the first step, in order to lower the impact on deformation caused by cutting. The roughness of the inner structure was first measured using the Surface Roughometer, and R_a_ was used here to represent the roughness value in this study. The sampling length was taken as 2.5 mm in accordance with JB/T 7051-2006 and the interval number was 5. The length between each interval was 1 µm. A Gaussian filter was used and the length of the accelerating and decelerating parts was 1.25 mm. All the positive and side faces were measured in the direction along the inner structure. Each surface was measured three times in this study to prevent error in the measuring process. The average value of each surface roughness for every specimen was calculated separately and used as the roughness of the positive face and the side face. Flatness was measured using the trilinear coordinates measuring instrument with a laser triangulation technique using the HP-L-20.8 Scanner Head provided by Hewlett-Packard Development Company, Palo Alto, CA, USA. The working distance of this scanner head was 180 ± 40 mm with a rate of 100 Hz. The minimum point distance was 0.013 mm, while the shape error was within 9 µm. Specimens were scanned first to obtain point cloud data with ultrafine quality. The direction of the laser probe was changed (vertical, horizontal, and 45°) in the scan process, and the focal spot was focused on the inner structure to increase the scan quality. Some noisy points were deleted from the point cloud data, and then the data were fitted with the imported three-dimensional design graphic. Specified surfaces were chosen after fitting, and the flatness value was obtained. No parameters were changed in this process, and all calculations were done by Polyworks, Version 2016 (InnovMetric Corporation, Québec, Canada).

The third step was to process the relevant data to make the trend clearer. The measured data were inserted in the table and line charts were generated in Origin, Version 8, OriginLab Corporation (Northampton, MA, USA).

The last step was to explain the measured data. The side and positive surfaces of the inner structure were interpreted by the 3D surface profilometer(RTEC, MFD-F profilometer 2207, San Jose, CA, USA). They were captured under interferometry objectives with a magnification of 20×. The numerical aperture was 0.4 while the working distance was about 4.7 mm. The field of view was about 860 µm × 650 µm while the spatial sampling was 0.34 µm. The resolution was about 0.35 µm. The scanning electron microscope (Carl Zeiss, Sigma300, Oberkochen, German) provided by Additive Manufacturing Products Supervision and the Inspection Center of Jiangsu Province was also used to capture the morphology of the side surface printed using two different scan strategies. The accelerating voltage was 20 kv while the working distance of this instrument was 8.7 mm. The magnification was 42× and the detector used here was SE2. The design model and measured position in this study are shown in Figure 2.

## 3. Results and Discussion

**Figure 3:**
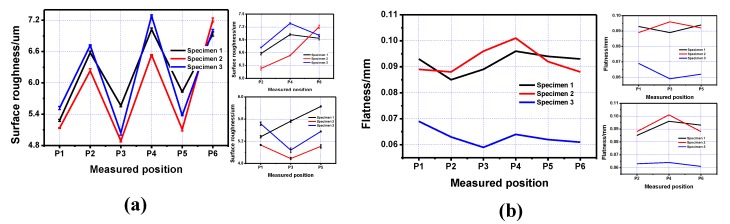
Line charts of roughness and flatness of the positive surface on the inner structure printed using three different scan strategies. (**a**) Positive surface roughness of the printed specimens; (**b**) Positive surface flatness of the printed specimens.

The measured data of the positive surface of the inner structure, including both roughness and flatness, are listed in Table 3. The line charts of the data are shown in Figure 3. The flatness value on the positive inner structure surface printed by the square-framed scan strategy was much lower than that printed by the other two scan strategies. The performance of the positive surface roughness printed using different scan strategies was similar according to our experimental data. The average roughness value for each specimen is listed in the table; however, there was a deviation between the measured data and the average value, which was about ±0.10 µm on the positive surface. We should note that although the roughness values of Specimens 1 (printed by Z-shape scan strategy vertical to the inner structure shown in Figure 1a), Specimen 2 (printed by Z-shape scan strategy along the inner structure shown in Figure 1b), and Specimen 3 (printed by square-framed scan strategy shown in Figure 1c) were almost the same, there was a different roughness performance on the upper-positive surface compared to the lower-positive surface. The roughness values of P1, P3, and P5 were lower compared to that of P2, P4, and P6, which means that the lower-positive surface roughness was better than the upper-positive surface roughness; this was mainly due to the fact that the coagulation of powder on the upper-positive surface was more significant than that on the lower-positive surface.

**Figure 4:**
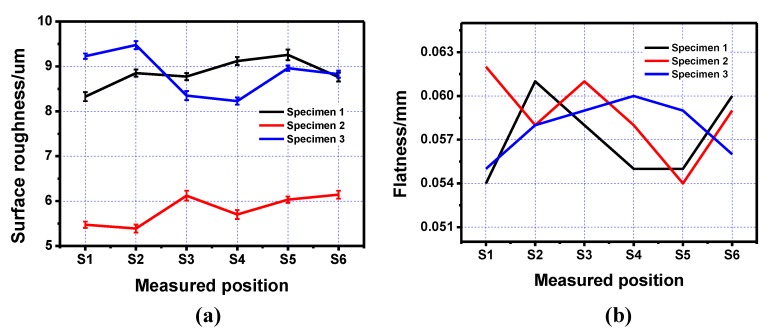
Line charts of roughness and flatness of the side surface on the inner structure printed using three different scan strategies. (**a**) Side surface roughness of the printed specimens; (**b**) Side surface flatness of the printed specimens.

A similar method was applied to the side surface quality of the inner structure. The measured data and line charts are shown in Table 4 and Figure 4. The inner structure part printed along the scan direction showed a much better side surface roughness property compared to that printed using the other two scan strategies. As for the side surface flatness, the specimen printed using this scan strategy did not show a significant improvement compared to specimens printed using the other two strategies. Another interesting point can be seen on the deviation between measured data and the average value of the side surface roughness. The deviation of side surface roughness was ±0.17 µm, which was almost twice compared to that on the positive surface. This was mainly caused by the randomization of the powder conglutination on the side surface.

**Figure 5:**
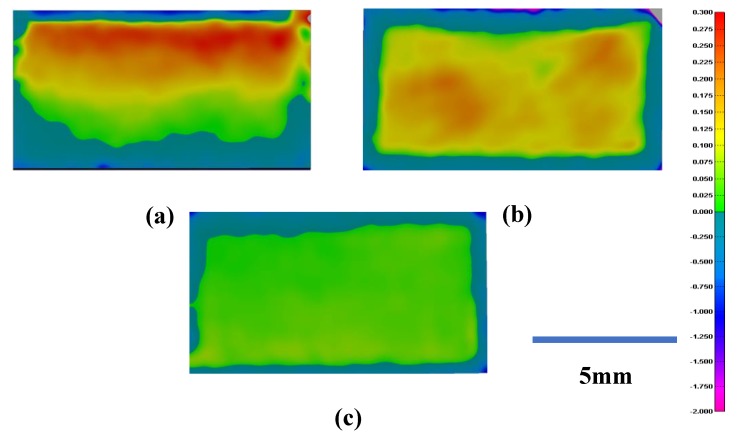
Bending deformation of the three specimens printed using three scan strategies: (**a**) the Z-shape scan strategy vertical to the inner structure; (**b**) the Z-shape scan strategy along the inner structure; and (**c**) the square-framed scan strategy.

According to the test results, the scan strategy showed an obvious impact on the inner structure surface quality. The different performances on positive surface flatness printed using these three scan strategies were mainly caused by the bending deformation of the specimens. The powder melted using the scan strategies for Specimens 1 and 2 had less time to cool down when the radiation of the laser increased the temperature again in a short time; this caused the curvature of the layer. To verify this, fitting images were taken to show the bending deformation of the specimens printed using these scan strategies, as shown in Figure 5. We should note that the specimens printed here were the traditional cubic specimens that were used to show the bending deformation of the specimens caused by the scan strategy.

The different colors shown in Figure 5 represent the deviation between the scan model and the design model. All the specimens were kept under the same scale bar, and the reference plane set here was the same as the height of the printed part. To prevent the bending deformation caused by wire cutting, these three specimens were not cut from the base plate. The red area means that the scan model was higher compared to the design model, while the blue area means the opposite. Just as shown in the scale bar, the deeper the color, the higher the deviation between the scan model and the design model. The specimens printed using the scan strategies vertical to and along the inner structure showed a significant deformation, while the specimen printed using the square-framed scan strategy had the lowest deformation in all three specimens. This result confirmed our explanation given before.

**Figure 6:**
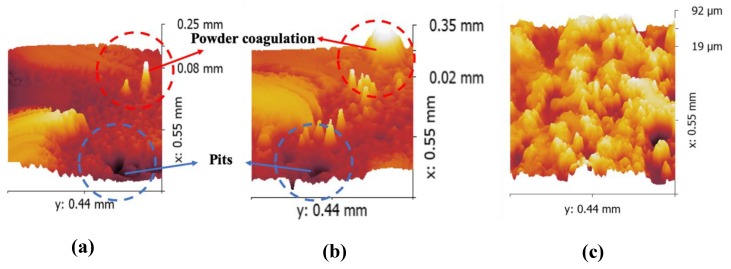
Side surface morphologies: Specimens 1 (printed using Z-shape scan strategy vertical to the inner structure) and 3 (printed using square-framed scan strategy) ((**a**,**b**), respectively); and Specimen 2 (**c**) (printed using Z-shape scan strategy along the inner structure).

As for the different roughness performance on the side surface of the inner structure, a 3D surface profiler was used to explain this phenomenon, as shown in Figure 6.

**Figure 7:**
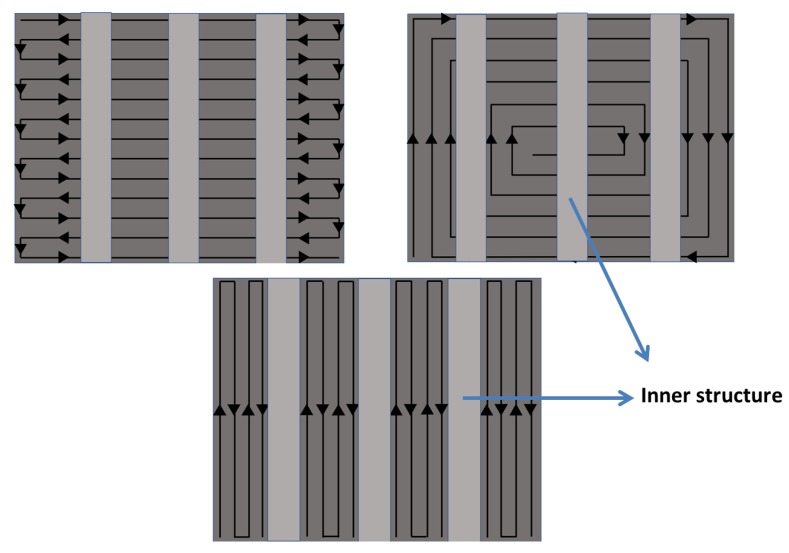
Specific scan strategies shown on the specimens with the inner structure.

The specimens printed using the Z-shape scan strategy vertical to the inner structure and the square-framed scan strategy had some obvious pits, as shown in Figure 6a,b, while the specimen printed using the Z-shape scan strategy along the inner structure had quite a smooth surface, as shown in Figure 6c. Surface roughness was highly affected by the presence of voids on the surface. In general, fewer pits on the surface led to lower surface roughness. When the inner structure did not print along the scan direction, the laser had to turn on and off frequently, which was difficult due to mode locking for the laser; this caused the frequency instability of the laser source and resulted in the temperature instability of the powder. This is the reason why the parts printed along the scan direction had a lower roughness on the side surface than those printed vertical to the scan direction (Figure 7).

**Figure 8:**
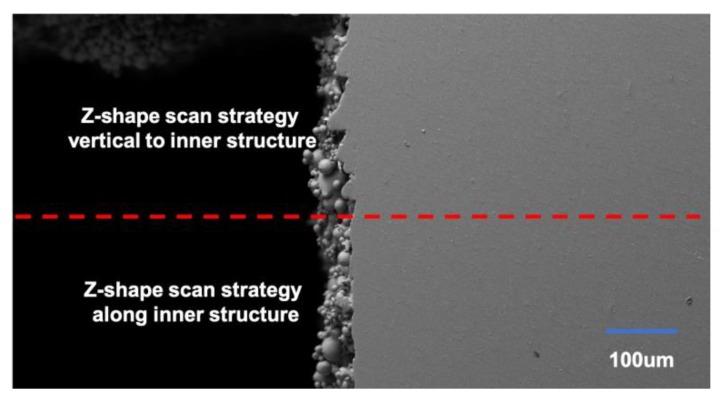
The morphology of the side surface printed using different scan strategies.

A scanning electron microscope was used to verify the explanation given here. To make the comparison clearer, the inner structure layer was printed using two different scan strategies. The lower half of the inner structure layers was printed using a Z-shape scan strategy along the inner structure while the upper half was printed using a Z-shape scan strategy vertical to the inner structure. The morphology of the side surface can be seen in Figure 8.

It can be seen clearly that the upper half had a significant extra coagulation of powder which led to the deformation of the side surface. As for the lower half, although the coagulation of powder was quite significant, its morphology was much better compared to the upper half.

**Figure 9:**
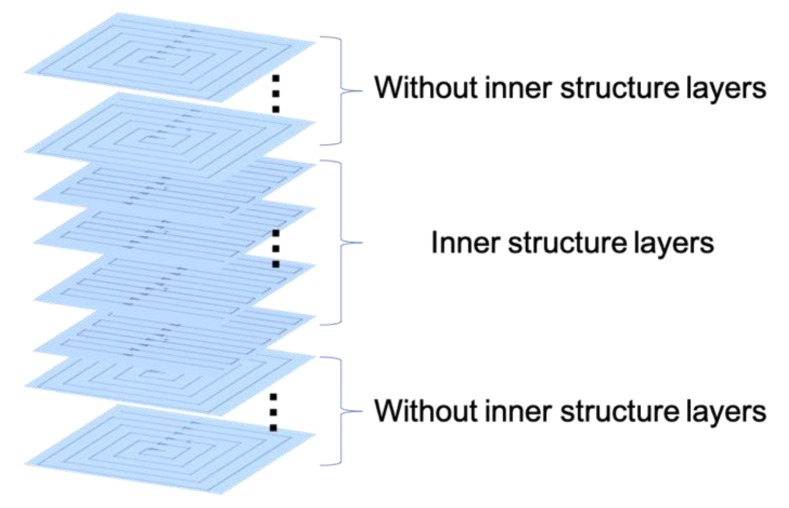
New scan strategy used in this study.

**Figure 10:**
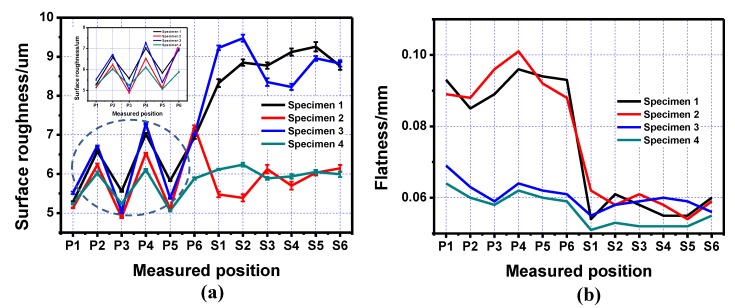
Line charts of roughness and flatness on the positive and side surfaces of the inner structure, printed using three different scan strategies and the modified scan strategy (Specimen 4). (**a**) Surface roughness of the four specimens; (**b**) Surface flatness of the four specimens.

Combining all the experimental data and test results, a new scan strategy was proposed for the inner structure part. First, the layers under the inner structure were printed using a square-framed scan strategy. The inner structure part was printed using a Z-shape scan path along the inner structure and the upper layers without inner structure were printed using a square-framed scan strategy, as shown in Figure 9. The final results showed that this specimen had the best performance out of all the specimens, as shown in Table 5 and Figure 10.

From the line charts shown in Figure 10, it can be clearly seen that the new scan strategy showed better overall performance compared to the other three specimens. The surface roughness of Specimen 4 was almost the same as the value of Specimen 2, while its flatness reached the level of Specimen 3. In another words, the specimen printed using the new scan strategy combined the advantages of the Z-shape along the inner structure and the square-framed scan strategies. This new proposed multiple scan strategy was more suitable for printing a specimen with an inner structure compared to the single scan strategy.

## 4. Conclusions

In this study, the relationship between scan strategy and surface quality of the inner structure was systematically studied. We found that:

1. The square-framed scan strategy showed a great improvement of the flatness of the positive inner structure surface and led to less bending deformation while printing.
2. The scan strategy printed along the inner structure improved the side surface roughness of the inner structure as the laser power was relatively stable.
3. Combining these two findings, a new scan strategy was proposed and improved the surface quality of the inner structure on both the side and positive faces.

## Future Work

As the bending curvature will occur during the printing process, more work is needed to find the maximum printing length of the inner structure.

## Figures and Tables

**Table 1:** Process parameters used in this study.

Parameter	Laser Power (w)	Laser Scan Velocity (m·s^−1^)	Layer Thickness (mm)	Hatch Space (mm)	Protective Gas
Value	180	1	0.05	0	Argon

**Table 2:** Relevant information about the powder used in this study.

Powder	D_10_ (µm)	D_50_ (µm)	D_90_ (µm)	O (wt.%)	N (wt.%)	H (wt.%)	C (wt.%)	Al (wt.%)	V (wt.%)	Fe (wt.%)
Ti6Al4V	27.7	37.0	49.0	0.175	0.024	0.0064	0.011	5.74	3.83	0.05

**Table 3:** Roughness and flatness on the positive surface of the inner structure part.

	Specimen 1		Specimen 2		Specimen 3	
	Roughness (µm)	Flatness (mm)	Roughness (µm)	Flatness (mm)	Roughness (µm)	Flatness (mm)
P1	5.281	0.093	5.133	0.089	5.517	0.069
P2	6.579	0.085	6.231	0.088	6.719	0.063
P3	5.563	0.089	4.888	0.096	5.040	0.059
P4	7.022	0.096	6.534	0.101	7.283	0.064
P5	5.829	0.094	5.108	0.092	5.377	0.062
P6	6.936	0.093	7.205	0.088	7.005	0.061

**Table 4:** Roughness and flatness of the side surface on the inner structure.

	Specimen 1		Specimen 2		Specimen 3	
	Roughness (µm)	Flatness (mm)	Roughness (µm)	Flatness (mm)	Roughness (µm)	Flatness (mm)
S1	8.327	0.054	5.475	0.062	9.228	0.055
S2	8.851	0.061	5.388	0.058	9.475	0.058
S3	8.774	0.058	6.121	0.061	8.351	0.059
S4	9.120	0.055	5.702	0.058	8.228	0.060
S5	9.256	0.055	6.033	0.054	8.960	0.059
S6	8.763	0.060	6.143	0.059	8.828	0.056

**Table 5:** Surface quality on the inner structure printed using the modified scan strategy.

	Positive Surface		Side Surface	
	Roughness (µm)	Flatness (mm)	Roughness (µm)	Flatness (mm)
1	5.242	0.064	6.115	0.051
2	6.025	0.060	6.237	0.053
3	5.239	0.058	5.887	0.052
4	6.110	0.062	5.940	0.052
5	5.064	0.060	6.052	0.052
6	5.885	0.059	5.993	0.055

## References

[1] Boschetto A., Bottini L., Veniali F. (2017). Roughness modeling of AlSi10Mg parts fabricated by selective laser melting. J. Mater. Process. Technol..

[2] Carter L.N., Martin C., Withers P.J., Attallah M.M. (2014). The influence of the laser scan strategy on grain structure and cracking behaviour in SLM powder-bed fabricated nickel superalloy. J. Alloys Compd..

[3] Olakanmi E.O., Cochrane R.F., Dalgarno K.W. (2015). A review on selective laser sintering/melting (SLS/SLM) of aluminium alloy powders: Processing, microstructure, and properties. Prog. Mater. Sci..

[4] Cheng B., Shrestha S., Chou K. (2016). Stress and deformation evaluations of scanning strategy effect in selective laser melting. Addit. Manuf..

[5] Lu Y., Wu S., Gan Y., Huang T., Yang C., Junjie L., Lin J. (2015). Study on the microstructure, mechanical property and residual stress of slm inconel-718 alloy manufactured by differing island scanning strategy. Opt. Laser Technol..

[6] Mohanty S., Hattel J.H. (2015). Cellular scanning strategy for selective laser melting: Generating reliable, optimized scanning paths and processing parameters. Laser 3D Manufacturing II.

[7] Wan H.Y., Zhou Z.J., Li C.P., Chen G.F., Zhang G.P. (2018). Effect of scanning strategy on grain structure and crystallographic texture of Inconel 718 processed by selective laser melting. J. Mater. Sci. Technol..

[8] Ahrari A., Deb K., Mohanty S., Hattel J.H. Multi-objective optimization of cellular scanning strategy in selective laser melting. Proceedings of the 2017 IEEE Congress on Evolutionary Computation (CEC).

[9] De Wild M., Schollbach T., Schumacher R., Schkommodau E., Bormann T. (2013). Effects of laser parameters and scanning strategy on structural and mechanical properties of 3D NiTi implants fabricated with selective laser melting. Biomed. Eng..

[10] Xie J.W., Fox P., O’neill W., Sutcliffe C.J. (2005). Effect of direct laser re-melting processing parameters and scanning strategies on the densification of tool steels. J. Mater. Process. Technol..

[11] Jhabvala J., Boillat E., Antignac T., Glardon R. (2010). On the effect of scanning strategies in the selective laser melting process. Virtual Phys. Prototyp..

[12] Parry L., Ashcroft I.A., Wildman R.D. (2016). Understanding the effect of laser scan strategy on residual stress in selective laser melting through thermo-mechanical simulation. Addit. Manuf..

[13] Rashid R., Masood S.H., Ruan D., Palanisamy S., Rahman Rashid R.A., Brandt M. (2017). Effect of scan strategy on density and metallurgical properties of 17-4ph parts printed by selective laser melting (slm). J. Mater. Process. Technol..

[14] Ghouse S., Babu S., Van Arkel R.J., Nai K., Hooper P.A., Jeffers J.R. (2017). The influence of laser parameters and scanning strategies on the mechanical properties of a stochastic porous material. Mater. Des..

[15] Calignano F., Lorusso M., Pakkanen J., Trevisan F., Ambrosio E.P., Manfredi D., Fino P. (2017). Investigation of accuracy and dimensional limits of part produced in aluminum alloy by selective laser melting. Int. J. Adv. Manuf. Technol..

[16] Mumtaz K., Hopkinson N. (2009). Top surface and side roughness of Inconel 625 parts processed using selective laser melting. Rapid Prototyp. J..

[17] Tian Y., Tomus D., Rometsch P., Wu X. (2017). Influences of processing parameters on surface roughness of HastelloyX produced by selective laser melting. Addit. Manuf..

[18] Bagheri Z.S., Melancon D., Liu L., Johnston R.B., Pasini D. (2017). Compensation strategy to reduce geometry and mechanics mismatches in porous biomaterials built with Selective Laser Melting. J. Mech. Behav. Biomed. Mater..

